# Children with palliative care needs – the landscape of the nordic countries

**DOI:** 10.1186/s12904-024-01447-x

**Published:** 2024-05-08

**Authors:** Anette Winger, Heidi Holmen, Dröfn Birgisdóttir, Camilla Lykke, Malin Lövgren, Mette Asbjoern Neergaard, Marika Grönroos, Johanna Kero, Oddný Kristinsdóttir, Ásta Bjarney Pétursdóttir, Charlotte Castor

**Affiliations:** 1 Department of Nursing and Health Promotion, Oslo Metropolitan University, St. Olavs Place, Post Box 4, 0130 Oslo, Norway; 2 Division of Technology and Innovation, Intervention Centre, Oslo University Hospital, Oslo, Norway; 3 Department of Clinical Sciences Lund, Faculty of Medicine, The Institute for Palliative Care, Lund University, Lund, Sweden; 4 Region Skåne, Lund, Sweden; 5 Section of Palliative Medicine, Department of Oncology, Copenhagen University Hospital, Copenhagen, Denmark; 6 Department of Oncology and Palliative Care, North Zealand Hospital, Hillerød, Denmark; 7 Department of Health Care Sciences, Palliative Research Centre, Marie Cederschiöld University, Stockholm, Sweden; 8 Advanced Pediatric Homecare, Astrid Lindgren Children’s Hospital, Karolinska University Hospital, Solna, 171 64 Sweden; 9 Palliative Care Unit and Child & Youth Palliative Care Team, Department of Oncology, Aarhus University Hospital, Aarhus, Denmark; 10 Department of Clinical Medicine, Aarhus University, Aarhus, Denmark; 11 Department of Pediatric and Adolescent Medicine, Turku University Hospital, Turku, Finland; 12 Welfare and Health, Satakunta University of Applied Sciences, Pori, Finland; 13 Pediatric department, Landspitali University Hospital of Iceland, Reykjavik, Iceland; 14 Faculty of Nursing and Midwifery, University of Iceland, Reykjavik, Iceland; 15 School of Health Sciences, Faculty of Graduate Studies, University of Akureyri, Akureyri, Iceland; 16 Department of Health Sciences, Faculty of Medicine, Lund University, Lund, Sweden

**Keywords:** Nordic countries, Pediatric palliative care, Care models, Research, Pediatric palliative education, Collaborative overview

## Abstract

**Background:**

To strengthen palliative care for children in the Nordic countries, an updated status of current needs, resources, clinical services, education, and research is necessary to align and consolidate future research. A Nordic research collaboration initiative for children with palliative care needs was assembled in 2023. Building on this initiative, this paper presents an overview of pediatric palliative care (PPC) in the Nordic countries’ (a) population characteristics, (b) care models and setting of care, (c) education and training, and (d) research.

**Methods:**

The Nordic initiative researchers collaboratively gathered and assessed available data on the characteristics of PPC within Denmark, Finland, Greenland, Iceland, Norway, the Faroe Islands, Sweden, and Åland. Data were compiled in a matrix with population characteristics, models- and setting of care, education and training, and areas of research in a Nordic context. The findings are narratively and descriptively presented, providing an overview of Nordic PPC.

**Results:**

In total, the Nordic child population comprises around six million children (0–19 years), of which about 41.200 are estimated to be living with a life-limiting and/or life-threatening condition. Healthcare services are provided through various care models, ranging from specialized care to homecare settings. Overall, there remain few opportunities for education and training with some exceptions. Also, Nordic research within PPC has been shown to be a growing field although much remains to be done.

**Conclusion:**

This overview is the first outline of the current PPC in Nordic countries. Although some differences remain important to acknowledge, overall, the strengths and challenges faced within PPC in the Nordic countries are comparable and call for joint action to increase evidence, services, and education to better serve the children, families, and healthcare personnel within PPC. Despite the varying structural premises for PPC, research endeavors aiming to provide evidence in this field seem increasing, timely and relevant for the Nordic countries, as well as the international context.

## Introduction

Pediatric palliative care (PPC) is currently under increasing focus in both healthcare and research in the Nordic countries due to increasing awareness. However, a lack of fundamental data or estimates regarding the child (0–18 years) population living with life-limiting and/or life-threatening (LL/LT) conditions remain across the Nordic countries, although international research suggest experiences within PPC that resonate well [1–3]. Being a child living with a LL/LT condition may be challenging for the child and family [3], and for healthcare services as well [4]. Life-limiting conditions include those where a premature death is expected because of the lack of a reasonable cure, while life-threatening conditions include those conditions where there is a high probability of premature death, but where there remains a chance of survival into adulthood [5]. Thus, PPC comprises conditions both with and without curative treatments [6]. To provide global standards for PPC accounting for global differences, an international expert group released a set of standards for PPC in 2008, which was recently revised [5, 7]. These standards comprise (1) clinical, developmental, psychological, social, ethical, and spiritual needs; (2) end-of-life care; (3) care models and settings of care; (4) PPC in humanitarian emergencies; (5) care tools; and (6) education and training for healthcare providers that aim to support PPC on a global scale [5]. These standards are well known also within the Nordic PPC context, in which there is a high degree of relevance to clinical care and research in what is a rather young research field [8].

Precise estimates for the number of children with palliative care needs in the Nordic countries are lacking. Efforts have been made to describe the population through various initiatives, which have found that the number of children with palliative care needs varies around the world [9, 10]. Estimates from England show an increase in the number of children in need of palliative care [6], with almost six times higher numbers of children in need of palliative care in the Global South [9]. Estimates from the Nordic countries remain limited because published data only exist for the number of children dying within each country each year, including those dying in accidents or suicide [11]. More than 400 specific diagnoses are categorized within PPC, the largest groups comprising children with neurological conditions, congenital conditions and cancer [12]. This heterogeneity poses some challenges, particularly when describing and estimating the population in need of palliative care services. International policies state that healthcare services should offer palliative care from an interdisciplinary team of health care providers, and according to the child’s and families’ needs [5, 13, 14], because palliative care services can be associated with quality of life among children and families [15]. However, there remains uncertainty about whether the care reaches all those in need, which may be because of factors such as lack of knowledge about what palliative care or PPC is [16–18], myths and assumptions of PPC only being relevant at end-of-life as an example [19], and lack of resources and organizational structures [20].

The field of PPC in an international context is rapidly developing and is often related to improved medical treatments that allow more children to survive their first years. As a result, the development of services and care to provide palliative care throughout the child’s life span is necessary. Similarly, there has been an increase in research within PPC, both to address the population, and their care needs [21]. Furthermore, the development of services necessary to care for the child and the family in a location of their choosing has also been examined in the literature [3]. However, conducting research within this population comes with both possibilities and challenges because research can pose a burden on the participants while providing new knowledge [2, 14]. Research initiatives must align to clinical practice but ensure that the research is soundly and timely and conducted in a way that places as little burden as possible on the participants. This is particularly important in a field that is developing as rapidly as the PPC.

In the context of the Nordic countries, we anticipate similarities among the Nordic countries regarding their inhabitants and populations, their welfare systems, and the organization of healthcare services [22]. All Nordic countries have well-developed and (75–85%) publicly financed healthcare, with expenditure representing 8–9% of gross domestic product. Most healthcare is provided free of charge for inhabitants, with national consistent rules for patient charges. Life expectancy has increased significantly over the years in all countries, even though the population structure varies slightly, with Sweden having the oldest and Greenland having the youngest populations. The Nordic countries share similarities in the birthrates, and similar immunization programs, with minor differences in vaccination against tuberculosis and whooping cough, measles, and rubella. Furthermore, of relevance for health care and research regarding children is the strong commitment to children’s rights among Nordic countries [22]. The United Nations Declaration of the Rights of the Child, which defines children’s rights to protection, education, health care, shelter, and good nutrition [23], is ratified in all Nordic countries and is part of Norwegian, Danish, and Swedish national laws. Particularly, the rights to healthcare for children with palliative care needs include having access to services, being staffed by trained personnel, and adhering to the international standards for PPC [5].

Potentially, research collaborations among Nordic countries could be relevant within small fields. A Nordic collaboration might increase the conduct and relevance of future PPC research and implementation of new evidence, and the current lack of overview within Nordic PPC research calls for joint forces for stronger research. Thus, the current paper aims to present an overview of PPC in the Nordic countries regarding (a) population characteristics, (b) care models and setting of care, (c) education and training, and (d) research.

## Methods

### Design and process

**Fig. 1:**
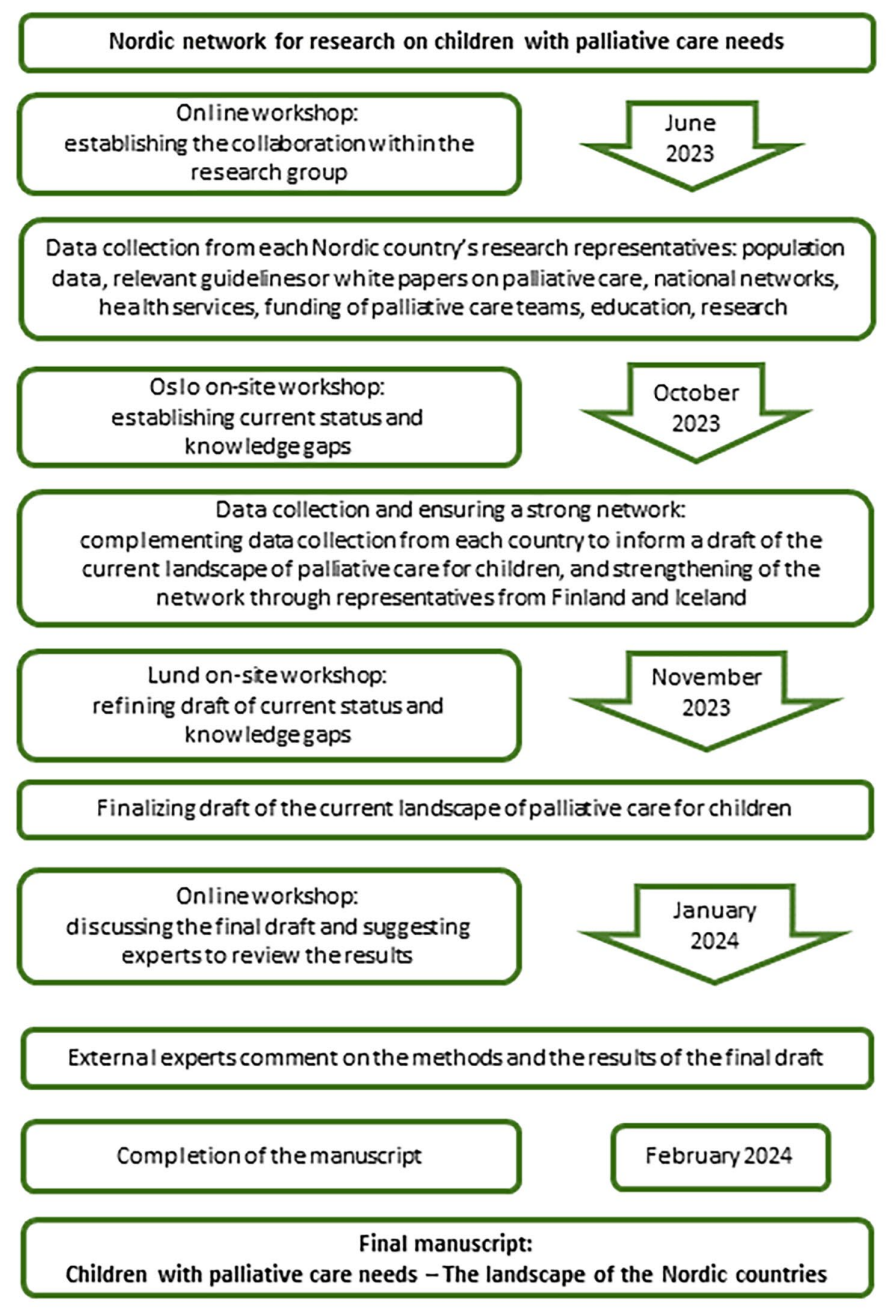
The process of constructing the Nordic PPC overview

This paper presents an overview of PPC from different perspectives in the Nordic countries, comprising Denmark, Finland, Iceland, Norway, Sweden, the autonomous territories of the Faroe Islands and Greenland, and the autonomous region of Åland. A Nordic research collaboration on PPC was initiated in the spring of 2023, with workshops held during the autumn of 2023 (Fig. 1). The initial research group consisted of seven Nordic experts in PPC from Institutes of Research and Higher Education and university hospitals in Denmark, Norway, and Sweden. An online workshop was held to introduce the participants and share visions of collaboration and reflect on the need for a status draft of the current Nordic landscape. The first workshop was followed by collection of data on relevant topics from each Nordic country, prior to an on-site workshop in Oslo, Norway. In this second workshop, the aim was to consolidate and discuss future collaborative work, in the short and long terms, regarding research in PPC. Data collected from each Nordic country was presented by the country’s representatives, and following a discussion, an overview with mapping of the PPC landscape in all the Nordic countries was deemed necessary and agreed upon by all participants. The third workshop took place in Lund, Sweden, to refine the data at hand and discuss the current overview of Nordic PPC. In addition, to strengthen the network and ensure all Nordic countries were represented with experts in the field, four more researchers were invited to this meeting. Two from Iceland and two from Finland participated in online discussions with the rest of the group, contributing information about PPC in their respective countries. Our fourth meeting took place online in January 2024, and the specific aim of this last meeting was to discuss the draft of the overview and align priorities with future research. In between the meetings, the group members were assigned work to prepare before each workshop. When a preliminary final draft of the Nordic overview was ready, it was sent to external Nordic experts in PPC for their voluntary comments. These experts comprised a group representing each Nordic country, with various experience in terms of clinical and academic competencies. In total, 11 experts provided comments on the results based on whether each section of the results was accurate, and, if not, their comments as to what was missing. The comments from these experts were then incorporated into the final results. All external experts are acknowledged in the Acknowledgements section.

### Data processing

Data collection for this overview was conducted in pairs of authors through online searches, obtaining information from our professional networks, and through personal knowledge about PPC in their country. Data from all the countries were compiled in a matrix informed by parts of the International Standards for PPC [5], consisting of information about population characteristics; models and setting of care; education and training; and research areas in a Nordic context.

Data on population characteristics was retrieved from the Nordic statistics database [11], which describe each country’s population and estimates on the number of children 0–19 years old. In addition, the website of the Nordic statistics projection was used for updated numbers [24]. Our results are reported based on the age span used in the Nordic statistics database for children between the ages 0 and 19 (from now referred to as children) compared with 0–18 years, as the age span for being a child or adolescent is often defined [23]. Based on these numbers and following the anticipated count of children with LL/LT conditions in England [6], we estimated the count and percentages of children with LL/LT conditions within each Nordic country using the estimate of 66/10.000 children having LL/LT conditions. Our calculations were based on child population data reported in the Nordic statistics database [11, 24]. Predicted future prevalence of children with LL/LT condition was calculated based on estimated numbers, also from the Nordic statistic database using the same estimates as for the current prevalence (66/10.000) (Table 1). Due to fewer children dying during childhood, the numbers of children with PC needs are expected to increase.

Descriptions of models and setting of care were made based on the expertise within the Nordic initiative in PPC and our conversations with other experts in the field. Formalization and acknowledgment of PPC in the Nordic countries is an ongoing process making comprehensive and yet consistent mapping impossible. Therefore, a summary of basic structures, organizational development, and trends, as identified in the later part of 2023, is described rather than reported in detail. Differences and similarities are described, and at times, single examples are provided.

The available education and training were initially described according to the common systems for higher education in Europe, following the structure of graduate level, postgraduate level, Ph.D. level, and other courses not at universities. Experts behind this overview developed a draft of existing educational activities within PPC, drawing on their knowledge of education in each Nordic country. Then, these drafts were shared and discussed in a meeting for the others to comments on and add educations or courses they knew of. Altogether, the authors of this current overview are affiliated with several educational institutions among the Nordic countries, and are experts within the field of PPC, often invited to teach various courses on a national, Nordic, and international scale. Furthermore, it was also shared with the selected external experts from each country that had the opportunity to add further information if needed. Thus, the overview on education did not require any additional systematic searches among educational institutions to identify further courses. Within the available education or courses, we sought to gather the scope of the learning exercise, either through the number of study credits (European Credit Transfer and Accumulation System – ECTS) or number of hours estimated for the course for comparability. In addition, the intended target group was identified.

Current areas of research were described by the expert group based on their insight. The experts from each country wrote a summary of the research status in their country before the summaries were revised into one condensed text on Nordic research. In addition, we searched for relevant research among peers, other experts, and relevant institutions and through reference searches in systematic reviews. This process was conducted to provide an overview, but not an exhausting review of all available evidence. Thus, we will reference research that constitutes some examples.

The following overview comprises a descriptive presentation of PPC according to the population characteristics; models and setting of care; education and training; and areas of research. The present study did not require ethics approval as the intention is to present an overview of the field and is only based on already published and available data.

## Results

### Characteristics of children in need of palliative care

Among the Nordic countries, Sweden has the largest population with 10.5 million inhabitants, and the largest child population, with almost 2.5 million children (Table 1). The child population (0–19 years) in the Nordic countries ranges from 21 to 28% of the total population. The Faroe Islands and Greenland stand out with the highest proportion of children with 28% and 27%, respectively.

**Table 1:** Data and estimates of the child population in the Nordic countries ^a^

	Denmark	The Faroe Islands	Finland	Greenland	Iceland	Norway	Sweden	Åland
**Population data**								
Total population ^b^	5,932,654	54,107	5,563,970	56,609	387,758	5,488,984	10,521,556	30,359
Children (0–19 years), n (%) ^c^	1,300,453 ^b^ (22)	14,918 (28)	1,150,078 (21)	15,069 (27)	79,578 ^d^(21)	1,236,596 (23)	2,439,823 (23)	6667 (22)
Child deaths (0–19 years) per year in 2022, n (%) of the child population	317 (0,02)	9 (0,06)	263 (0,02)	22 (0,15)	17 (0,11)^d^	249 (0,02)	492 (0.02)	NS ^e^
**Population estimates**								
Estimates of children with LL/LT conditions, n ^f^	8.583	98	7.591	99	525	8.162	16.103	44
Predicted future prevalence of LL/LT conditions by 2030, min-max. ^g^	8 713- 10 952	100- 126	7706- 9686	101- 127	533- 670	8285–10,415	16,347–20,548	45- 46

LL/LT life-limiting and/or life-threatening conditions; PPC pediatric palliative care

^a^ Populations as of January 1, 2023. Assumption of the size and age distribution of the population based upon assumptions regarding future fertility, migration, and death rates based upon the situation at present [11]

^b^ Numbers from January 1, 2023. Projected numbers based on different dates and year ranges from 2020–2023 from each country (total population are retrieved from Nordic statistics dataset)

^c^ (%) of the total population in each country

^d^ (0–18 years)

^e^ Not specified for Åland, N reported in numbers from Finland

^f^ Estimates of 66/10.000 children in need of PPC based on the child population as of January 1, 2023 [6]

^g^ Predicted future prevalence of LL/LT conditions ranged from 67.0 (95%CI 67.7–66.3) to 84.22 (95%CI 78.66–90.17) per 10,000 by 2030

**Fig. 2:**
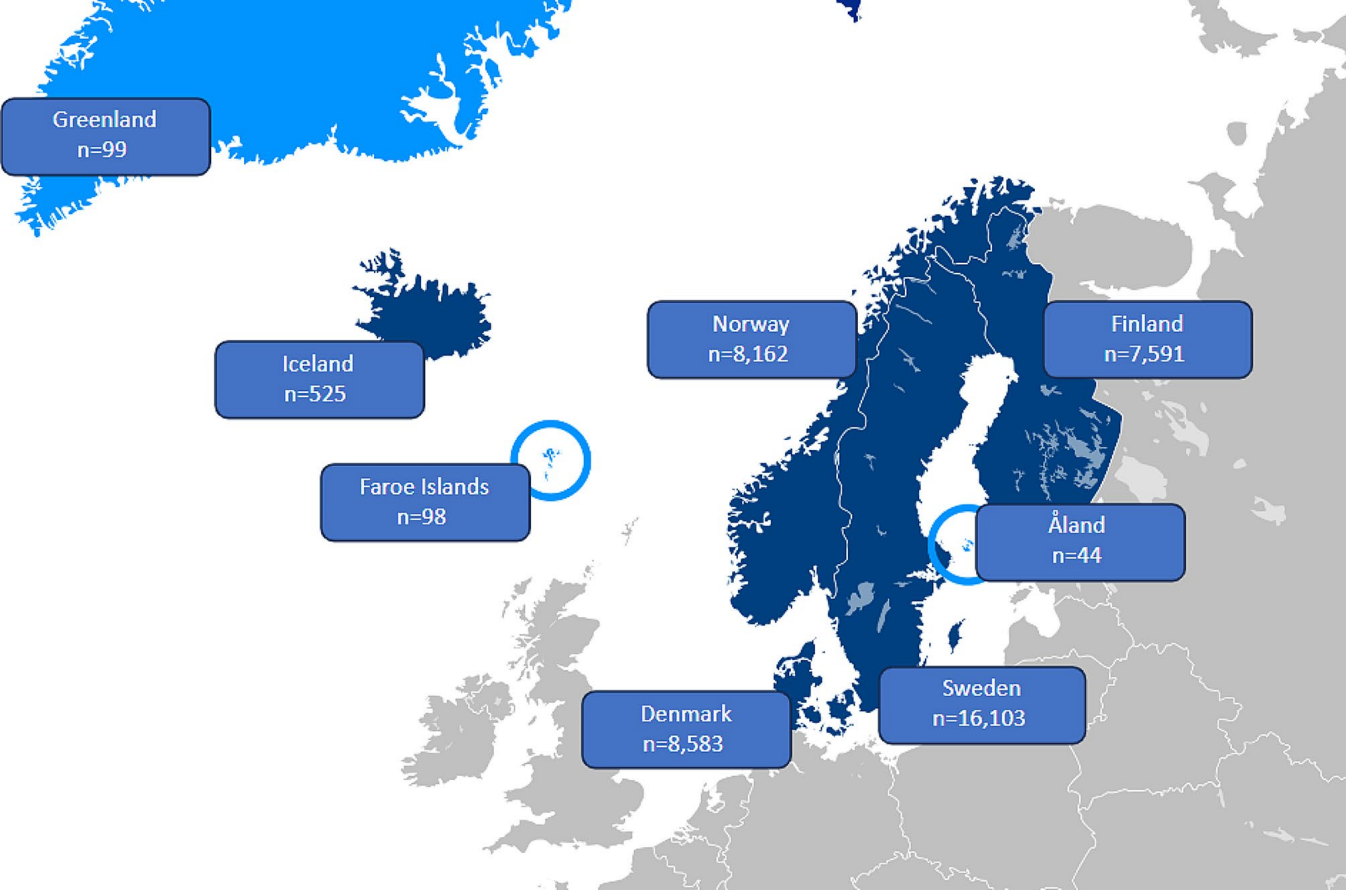
Estimates of children (0–19 years) with palliative care needs in each Nordic country in January 2023, based on Fraser et al. 2021 [6]

Even though the Nordic countries have well-developed health statistics for specific diagnosis or conditions, none of the Nordic countries have specific statistics comprising one register for PPC that cover all LL/LT conditions that affect children. As a result, no fundamental statistics exist for children with LL/LT in the Nordic countries. The number of children in need of palliative care is estimated to be equivalent to around 41,200 children living with LL/LT conditions in Nordic countries. The total number of children dying each year is small in all the Nordic countries and includes children dying from other conditions than LL/LT, such as accidents and suicides. Altogether, the Nordic child population consists of more than six million children (0–19 years), of which only 0.03% die each year. The estimates of children (0–19 years) in need of palliative care in each country are shown in Fig. 2.

### Care models and settings of care

Variations in the recognition and understanding of PPC have implications for healthcare provision in the Nordic countries, leading to variations both between countries or geographical areas and between different care organizations. Over time, the focus on defining the levels of PPC and on end-of-life care has changed towards recognizing the need for early and continuous integration of PPC as part of all care of children with LL/LT. In Denmark and Norway, the national authorities have provided guidelines for PPC, while in Sweden, children are about to be included in the revised National Health Association guideline for palliative care, which previously only includes end-of-life care for adults. Furthermore, PPC is one of several focus areas of care that have been identified as nationally prioritized by the authorities in Sweden. In Finland, the Ministry of Social Affairs and Health have given recommendations on palliative care and PPC services, and regional advance care plans are currently under revision to support the development of national guidelines preliminary being launched in 2025. In Denmark, Norway, and Sweden, national clinical guidelines are produced, and the implementation is ongoing. Åland follows Finland’s jurisdiction and PPC guidelines, while the Faroe Islands and Greenland follow Denmark’s jurisdiction and PPC guidelines. In Iceland, informal collaborations between healthcare facilities regarding palliative care compensate for the lack of national guidelines regarding PPC. In Denmark and Norway, the national government has dedicated funding for the development of specialized PPC on equal terms. In Sweden, local and regional self-governing means that national investments are prioritized independently so that PPC in some geographic regions have a more or differently developed PPC than other regions.

Most of the care for children with LL/LT conditions is provided as part of the publicly financed healthcare. Thus, the care models and settings of care for these children are carried out as multiprofessional care for the whole family at wards and facilities specializing in pediatrics, being supplemented by services for providing homecare, PPC consultations, or hospice care. PPC is sometimes organized with different levels of formal and informal collaboration with adult palliative care facilities.

Homecare services have been and are being developed continuously within several Nordic countries. Because of structural, traditional, and variance in geography and population density, with both large areas of sparsely populated areas and densely populated cities, countries have focused on different versions of homecare services. In some areas, homecare services are provided as hospital-based home services organized from a pediatric service, reaching from single hours per week to 24/7 access. In other areas, homecare is provided by community services, which is often specialized mainly in adult care. Often, for a child to receive PPC at home, a collaboration between community healthcare providing ongoing care and hospital-based services for procedural services is required. However, although homecare is offered throughout the child’s illness trajectory in some areas, it is offered exclusively for children receiving end-of-life care in other areas. Single pediatric hospices or pediatric palliative in-patient care wards are offered in Denmark, Norway, and Sweden, and in Iceland a care facility offering respite care for children with LL/LT is available, however, such services often reach a limited number of Nordic children in need of PPC.

Furthermore, in each of the health regions in Norway (*n* = 4) and Denmark (*n* = 5), health services are obliged to provide local and/or regional PPC teams, and in Finland, all five university hospitals provide the same. Regional PPC teams in Norway and Denmark are located at university hospitals, and team members include clinicians from different disciplines and professions, with years of experience with children with LL/LT conditions and their families. Their mandate is to build and provide knowledge, guidance, and support for healthcare personnel in their patient care. In addition, some regional teams also have a local role in which they provide first-hand PPC. In Iceland, informal collaboration is arranged together with adult palliative care teams according to each child’s needs. In Sweden, the provision of PPC consultations varies in a range from locally or regionally organized PPC teams from pediatric or palliative care services to no formal services focusing on PPC.

In all Nordic countries, multiprofessional habilitation and rehabilitation services for children with LL/LT are available for children in need of such services. Although they are often organized in different ways, they are commonly provided as part of hospital based pediatric neurology and within primary care or as residential institutions for children. The estimated most common place of childhood deaths is in a hospital, with variations in relative percentage depending on country, illness and age of the child, as well as over time. In general, there is no exact information about place of death for children in need of PC in the Nordic countries. A Danish study found that in 2011–2014, 89% of all children dying, died in a hospital, 5% died at home and 6% died other places than hospital or home. Further the study demonstrated a decrease of home deaths for these years compared with the period of 1994–1998 [25].

To collect data regarding those in need of palliative care, some registry initiatives exist. In Denmark, there is a recommendation to register all children affiliated with specialized PPC teams in the Danish Palliative Care Database, with quality indicators reported yearly. Sweden has a national quality register covering the symptom burden and care over the last week of life, with a total coverage of about 50% of all deaths, but currently, less than 10% of these deaths come from the child population.

### Education and training in palliative care for children

With only a few exceptions, formal education in PPC is scarce and fragmented and generally lacking in all levels of education systems in all Nordic countries (Table 2). As part of the implementation of the national guidelines for PPC in Norway [26], an educational program for nurses (master’s degree level) was established in 2017 and starting in 2018 the program became interdisciplinary. Sweden developed an interdisciplinary PhD course and a course on an advanced level in PPC in 2022. A Nordic specialist course in palliative medicine (NSCPM) for physicians was established in 2003 with participants from all Nordic countries, and PPC is included in their curricula on a regular basis, however the NSCPM ends in 2025 and each Nordic country has to educate physicians in palliative care locally.

Some single courses and informal lectures on PPC lasting for a few hours exist in some bachelor education programs and higher education throughout the countries but typically as part of courses on general palliative care without specification in the curriculum. Also, as part of specializations like, for example, medical doctors (MD), special courses are provided although with a rather brief focus on PPC as part of adult palliative care education programs. There are examples of stand-alone courses in PPC and the possibility of achieving an MD specialization in PPC (Table 2). However, the field of education in PPC is developing; for example, the Danish Pediatrics Society is currently working on a curriculum for expert approval in pediatric specialized palliative care. In the future due to shortage of health care professionals, we anticipate increased involvement of nurse assistants and health care workers providing vital parts of the care for the PPC population. While education in PC exists for this group, to the best of our knowledge there are currently no formal organized PPC training programs with standardized curriculum tailored for this personnel group in any of the Nordic countries.

**Table 2:** Formal education and course in Pediatric Palliative Care (PPC)

	Graduate level	Postgraduate level	Ph.D. level	Other courses (Not placed at the universities)
Denmark	No formal education on PPC ^a^	**MD**: One-day (out of 30 days) course in PPC at the new Danish education in palliative care. **MD**: Specializing in pediatrics includes one hour of PPC.	No formal education on PPC ^a^	**MD**: The Palliative care committee in the Danish Pediatric Society is working on a curriculum for expert approval in pediatric specialized palliative care. **Interdisciplinary**: PPC teams offer advice, guidance, and training to health and care staff working with children with PC needs.
The Faroe Islands ^b^	No formal education on PPC ^a^	No formal education on PPC ^a^	No formal education on PPC ^a^	No formal education on PPC ^a^
Finland	No formal education on PPC ^a^	No formal education on PPC ^a^	No formal education on PPC ^a^	**MD**: Special competence training on palliative medicine with own requirements for doctors working with children and adolescents. Organizations offer advice, guidance, and daily continuing professional education to health- and care staff working with children with PC-needs
Greenland**	No formal education on PPC ^a^	No formal education on PPC ^a^	No formal education on PPC ^a^	No formal education on PPC ^a^
Iceland	**RN**: One lecture in PPC at the University of Iceland	**RN**: One lecture in PPC in Intensive care nursing master’s degree program (MSc) at the University of Iceland	No formal education on PPC ^a^	**MD**: In collaboration with NSCPM
Norway	No formal education on PPC ^a^	**Interdisciplinary**: Part-time (30 ECTS) for healthcare professionals, social workers, and teachers in PPC.	No formal education on PPC ^a^	**MD**: A curriculum in PC for medical doctors is developed and the course has some hours dedicated to PPC. **Interdisciplinary**: Course in Palliative Care for Children for healthcare providers (ended). **Interdisciplinary**: PPC teams offer advice, guidance, and training to health and care staff working with children with PC needs.
Sweden	No formal education on PPC ^a^	**RN**: Specialist nursing in palliative care including PPC at Marie Cederschiöld University in collaboration with Sophiahemmet University. **MD**: Adjusted specialist training in palliative care allows specialization in pediatric palliative medicine. One MD completed; some are ongoing **Interdisciplinary**: A master program in caring sciences including an option for PPC focus at Marie Cederschiöld University **Interdisciplinary**: Freestanding course in PPC at Marie Cederschiöld University (7.5 ECTS).	**Interdisciplinary**: Freestanding course of 7.5 ECTS in PPC at Marie Cederschiöld University. **Interdisciplinary**: Freestanding course on 7.5 ECTS in palliative care including pediatric and adult care at Marie Cederschiöld University.	**Interdisciplinary**: Regional Cancer Centers in Sweden, in collaboration with Skåne University Hospital, have made a digital introduction (67 min) to PPC allaying with the national guidelines. This is further elaborated on in a second digital course made by Astrid Lindgren Pediatric Hospital. Both are available online free of cost. **Interdisciplinary**: Shorter (2–8 h) national, regional, and local courses and webinars are held throughout the year. **Interdisciplinary**: PPC consult teams offer advice, guidance, and training to health and care staff working with children with PC needs.
Åland ^c^	No formal education on PPC ^a^	No formal education on PPC ^a^	No formal education on PPC ^a^	No formal education on PPC ^a^

^a^ No formal education on PPC, but elements of PPC might be included in other formal courses; ^b^ with full access to all Danish education; ^c^ With full access to all Finnish education

ECTS - European Credit Transfer and Accumulation System; MD – medical doctor; PC – palliative care; PPC – pediatric palliative care; RN – registered nurse

National associations focusing on PPC are available in some countries, such as a pediatric section in the Association of Finnish Palliative Medicine, Danish Pediatric Society for Physicians Working with Palliative Care for Children, a special interest group for PPC in the Norwegian Pediatric Association since 2019, a multidisciplinary competence network for palliative care for children in Norway, and a multidisciplinary Swedish association for PPC. In addition, several other PPC focused networks between and within professions, organizations, regions, and illness-specific groups exist.

### Research on palliative care for children in the nordic context

Overall, the current Nordic research has mainly an illness-focused approach rather than a palliative care approach. Various research environments focus on children with an LL/LT condition such as oncology, neurology, cardiology, or rare diseases, without establishing that the research is conducted with the primary aim of being within an overarching understanding of PPC. However, this research concerning children with LL/LT conditions benefits those children who are eligible for PPC. Examples include research on symptom management for various conditions, homecare for children with various conditions, also including eHealth care models, and bereavement care.

Most studies from the Nordic countries are conducted as qualitative, epidemiological, or observational studies and aim to describe the population and the needs of the child throughout the illness trajectory [25], their families [3, 27–29], and healthcare professionals working within PPC [16, 17, 30]. Common designs include cross-sectional studies, registry-based studies, and qualitative interview- or focus-group studies. In addition, systematic or scoping reviews aiming to summarize evidence and identify knowledge gaps have been conducted [3, 21, 31–34]. Some studies exist regarding implementation and models of care interventions [35], aiming to support symptom management and family communication, and psychosocial support [36], and all countries have a research focus on homecare for children with palliative care needs [27, 30]. To provide data and enable longitudinal registry-based studies, some registries exist, such as those in Denmark [37] and Sweden [38]. Altogether, the range of interventions and implementations included in current or previous research remains limited among all Nordic countries even if good example exists [39–41].

In PPC, research is more often conducted with parents, siblings, or healthcare professionals rather than with children themselves; similarly, healthcare professionals are often included to share their experiences. Thus, a common limitation of previous Nordic research is the lack of ill children’s voices with their needs and experiences explained in their own words, which remains present in all research designs, with some exceptions [42, 43]. Ongoing research has a stronger emphasis on the inclusion of the ill children’s voices because they have a right to be heard.

To establish a clear direction for research in the Nordic countries, several initiatives have been made by researchers in a Nordic PPC context to systematically scope or summarize previous international research. This includes family [3, 44] and healthcare professionals’ experiences in home-based PPC [33], experiences with the organization of palliative care for children with LL/LT conditions and their families [3, 34], the use of eHealth in home-based PPC [31], current possibilities for children’s reporting of their own symptoms [32], and predictors for place of death among children [45]. In addition, a systematic review has focused on knowledge translation in PPC, finding that formalized education in a specialized setting is most often used to support knowledge translation [21].

## Discussion

The present paper has outlined the first overview of the current PPC in Nordic countries. We chose to structure the overview in terms of population characteristics; care models and settings of care; education and training; and research. This choice was based on the international standards in PPC [5], which act as a guide for optimal PPC. In our work, we found that these four are intertwined, with mutual consequences for how far the field has come in building robust evidence, services, or environments. Common to all is the lack of fundamental data and research to support the development of PPC. As a result, it might be challenging to develop standardized healthcare services for a population in which the needs and characteristics are diverse. Similarly, developing education and training for healthcare personnel when their tasks are unclear adds to the challenges in PPC. As a result, several small initiatives have been formed to address the population, their needs, proper care models and relevant education. Drawing on these initiatives and gathering and building upon this existing work is crucial for building a stronger evidence base in the future in Nordic countries. Regardless of the uncertainty of the estimates provided in this first overview of the Nordic PPC population, children in PPC require ongoing clinical care. Building on the existing evidence to further develop clinical care, evaluate new services, and provide evidence through research in this group remains crucial [14]. Thus, the potential of Nordic joint forces seems obvious, although basic structures in health services, clinical guidelines, and training vary among the Nordic countries.

The Nordic focus on the child as the natural center of care, as supported by the UN Convention on the Rights of the Child [23] and the understanding of PPC as a holistic process following the WHO’s definition and international standards [5] is unique. Within this understanding, there is also an emphasis on allowing the child to receive PPC at home rather than at the hospital if that is what the child wants [3], according to their right to be heard [23]. This increasing attention to homecare is seen among all Nordic countries. Altogether, the Nordic focus on children’s rights is also why the Nordic countries are a good fit for collaborative actions. Research endeavors must account for relevant differences but at the same time acknowledge the potential following the possibilities of increased clinical and research competencies in larger samples, which can offer more power when developing, testing, evaluating, and implementing interventions in a complex environment such as PPC [46].

A common problem in PPC, with consequences for this Nordic overview, is the various understanding and often nonexisting uses of the term PPC [16, 17, 47]. The often-heterogeneous understanding and use of PPC as a term makes it challenging to identify evidence regarding the child population in need of palliative care. Among health services, this poses a challenge because the inconsistent use of concepts and terms for the various models of care can cause confusion. Thus, the intention and meaning of PPC and its associated terms should be acknowledged when comparing populations and describing and transferring models of care. To decrease the risk of misinterpretation, a clear description of the content of care – rather than of the format – should be encouraged for both scientific and clinical writing. To further strengthen the role of PPC in the research, we urge researchers in the field of children with LL/LT to position their study in a PPC perspective. One way would be to reflect on how the research might relate to the three core domains of a palliative approach, described by Touzel and Shadd as “mortality acknowledge”, “whole-person care,” and “focus on quality of life” [48].

The proportion of children relative to the overall population in each country in percentage is similar and comparable. Acknowledging the burden that research can pose on the individual participants [2, 49], building evidence through Nordic research collaborations can potentially decrease the burden on the individuals because the research can be more robust when the population one is sampling from increases. This is particularly relevant when trying to describe and characterize the PPC population and their needs. Challenges regarding the estimates of children in need of palliative care are not unique in the Nordic countries; rather, they have been described before [6, 10]. Building upon already developed estimates can reduce the uncertainty regarding these estimates and provide more accuracy when developing care models and education to serve the population and its carers. At the same time, further research on percentages with specific PPC needs on a national population could be valuable because some differences in relation to, for example, abortion policies and age of the mother at childbirth from country to country occur [11].

The lack of fundamental data on population characteristics and needs may have consequences for the fragmented range of care models and settings of care. This overview highlights the range of healthcare settings and models of care that are offered throughout the Nordic countries, even though essential services for pediatric care are common in all Nordic countries. There seems to be a lack of research concerning the implementation of PPC teams supporting palliative care for children throughout the illness trajectory, despite the increased focus on PPC teams throughout the Nordic countries. The joint focus on PPC teams allows for comparison of care between areas, while at the same time, it is challenged by the formalization of the implemented teams, where some areas such as Denmark and Norway, have had more time in the formalization and standardization of their teams. At the same time, local initiatives in all countries have services that are well-established with high competence, without these teams being a formalized service acknowledged at the national level. Building on both the informal and formal teams might provide valuable knowledge for building robust teams among the other Nordic areas. Although efforts are made to provide care through a multidisciplinary team of health care providers, in accordance with the definition of PPC, this is not always the case. This is probably due insufficient knowledge about the philosophy of PC, limited of availability of personnel with relevant competency, conflicting priorities, or lack of resources in general. Throughout Europe there is a need for training, personnel, financial resources, and guidelines for PPC [50], and barriers to implementation of PPC is suggested to be addressed.

Because sparse formal PPC education is available in the Nordic countries, formal competence in PPC is limited compared with informal and clinically based competence through clinical learning. Also, there remains limited research concerning the development, effects, or experiences with education and/or training in PPC at any level of the educational system in the Nordic countries. Furthermore, at times being a minor subgroup of general palliative care, there is a risk of children as patients and children as relatives are grouped together which might challenge the identification of actual PPC involvement [51]. Often, recommended education in palliative care has three levels, from the basic competencies in the palliative approach to general, and finally, more specialized palliative care [52]. In the Nordic countries, most PPC seems to be given by healthcare staff specialized in either pediatric care or palliative care, rather than PPC as a unique field of expertise. Examples of how a stronger collaboration between pediatric and palliative care can be provided are documented, providing suggested benefits for the child’s quality of life [26, 53]. In addition, the effect of such collaboration from different perspectives is positive [29, 54, 55]; however, knowledge on how to optimize PPC to respond to the needs of the family is still limited within the Nordic countries. Recommendations suggest specialized education and training in PPC as an ongoing task [56]. Thus, the few opportunities for formal training in PPC hold as a strong argument for increased collaboration and sharing of existing knowledge from a clinical and scientific viewpoint, which would be timely and efficient. One example is the postgraduate education positioned in Norway, which accept students from the Nordic countries upon application and where applicants from the Faroe Islands have completed their PPC training. There exist several examples within palliative care for adults of collaborative efforts and joint forces among the Nordic countries in the premature phase of building a strong clinical and research field. To meet international recommendations for palliative care and overcome the shortage of physicians with experience and education in palliative medicine, NSCPM was established in 2003 [57]. This collaboration has been evaluated as a success in influencing the development of palliative medicine in Nordic countries [58]. However, because of the growing specialization in palliative medicine in the Nordic countries, the NSCPM courses will now end. This shows how Nordic collaborations facilitate an efficient use of resources, while building competencies and evidence in a Nordic collaboration. This might serve as an example to consider, especially in a small area such as PPC, that could be successful in a multiprofessional context.

### Limitations

This overview has several limitations. The lack of detailed fundamental data to support the overview of PPC in Nordic countries is a limitation. The current overview is based on the belief that the population of the Nordic countries is comparable to the English population of children with LL/LT conditions, and although the countries have obvious similarities, there might also be variations. One specific limitation with this is the rough division into age groups, leading to individuals aged 18 and 19 being included in this overview in terms of population, even though the description of care settings covers those for children 0–17 years of age. The population of children living with an LL/LT condition is greatly affected by each country’s regulations and clinical practices in relation to assisted reproduction and abortion. These vary significantly among countries, with Denmark having the highest level of assisted reproduction technologies for live births. Although medical development might lead to more children surviving, the birth rates are decreasing, and the population is getting older. Thus, the projected estimated future proportions of children in need of palliative care might be interpreted with caution. Furthermore, differences in national coding practices challenge the possibilities for comparisons between countries. For example, when causes of death were recorded as two or more on the death certificate, International Statistical Classification of Diseases and Related Health Problems (ICD) rules can be interpreted differently, leading to different choices of the underlying cause of death. Cultural differences in the reporting of certain conditions may also influence comparability [11]. There also seem to be differences between the countries in the use of concepts related to PPC, such as what entails hospice, homecare, or even specialized services, making comparability challenging. Such variations in concepts can challenge the development and testing of interventions if not accounted for through finding common ground. Finally, the much-bespoken international standard for PPC highlights the needs of migrated children with LL/LT conditions [5]. All Nordic countries have welcomed migrated families during the past few decades, but the number of children with LL/LT conditions among these specific populations is not covered in this overview, nor are they described in the current data material.

## Conclusion

The present overview is the first outline of the current PPC in Nordic countries. Overall, challenges faced within PPC in the Nordic countries are comparable and call for joint action to increase evidence, services, and education to better serve the children, families, and healthcare personnel within PPC. Despite the varying structural premises for PPC, research endeavors aiming to provide evidence in this field have been increasing, timely and relevant for the Nordic, but also the international, context. Furthermore, the present paper highlights such variances and equalities within and between countries that might be important to acknowledge when designing future research projects. To improve PPC in the Nordic region, we support an initiative to that is currently being launched, inviting stakeholders from all countries to collaborate on action plans addressing gaps in knowledge, education, care provision and research in PPC.

## Data Availability

The datasets generated in the overview will be made available upon reasonable request to the first author, Anette Winger. Open sources are cited accordingly in the reference list.

## Abbreviations

ECTS: European Credit Transfer and Accumulation System
ICD: International Statistical Classification of Diseases and Related Health Problems
LL/LT: Life-limiting and/or life-threatening
MD: Medical doctor
NSCPM: Nordic specialist course in palliative medicine
PPC: Pediatric Palliative Care
QL: Quality of life
PC: Palliative care
PPC: Pediatric palliative care
RN: Registered nurse
